# lncRNA CASC7 contributes to the progression of LPS- induced liver injury by targeting miRNA-217/TLR4 axis

**DOI:** 10.17305/bb.2024.10543

**Published:** 2024-07-26

**Authors:** Chengqin Sun, Yan Chen, Zhonge Chen, He Wang, Weiwen Yang, Xiaoqian Zhou

**Affiliations:** 1 Department of Gastroenterology, The First People’s Hospital of Gui Yang, Guiyang, Guizhou, China; 2 Department of Endocrinology, Baoding First Central Hospital, Baoding, Hebei, China; 3 Department of Gastroenterology, The Second People’s Hospital of Gui Yang, Guiyang, Guizhou, China

**Keywords:** Sepsis, liver injury, cancer susceptibility candidate 7 (CASC7), microRNA-217 (miR-217), toll-like receptor 4 (TLR4)

## Abstract

It has been reported that long non-coding RNAs (lncRNAs) are involved in sepsis-induced liver injury, while the role of cancer susceptibility candidate 7 (CASC7) in liver injury induced by sepsis remains elusive. In our study, 62 patients and 55 healthy controls were enrolled from our hospital, from whom CASC7 and microRNA-217 (miR-217) in serum samples were detected by quantitative real-time PCR (qRT-PCR). Then the sepsis-induced liver injury mice model was established by lipopolysaccharide (LPS). The effect of CASC7 on liver injury induced by sepsis was confirmed by hematoxylin and eosin (HE) staining, ELISA assay, transferase dUTP nick end labeling (TUNEL) assay, Annexin V-FITC apoptosis assay, and cell counting kit-8 (CCK-8) assay, respectively. Besides, RNA pull-down, luciferase reporter gene assay, qRT-PCR, and western blot were used to evaluate the underlying mechanisms. In this study, lncRNA CASC7 was significantly increased while miR-217 was significantly decreased in patients with sepsis-induced liver injury compared with that in healthy controls. There was a negative association of CASC7 and miR-217 in serum samples from patients with sepsis-induced liver injury and healthy controls. CASC7 was upregulated in a time-dependent manner in the liver tissues of LPS-treated mice. It was found that knockdown of CASC7 reduced the liver injury induced by LPS in mice. In vitro, LPS treatment enhanced cell apoptosis, while knockdown of CASC7 inhibited the role of LPS in cell apoptosis. Moreover, the knockdown of CASC7 suppressed the LPS-enhanced tumor necrosis factor-alpha (TNF-α) and interleukin 1 beta (IL-1β) expression. In addition, miR-217 was found to be a target of CASC7, and miR-217 mimic could reverse CASC7-promoted liver injury. Furthermore, toll-like receptor 4 (TLR4) was identified as the target of miR-217, and both CASC7 and miR-217 could downregulate the mRNA and protein level of TLR4. Additionally, TLR4 overexpression could reverse miR-217-inhibited or CASC7-promoted liver injury. Taken together, CASC7 contributes to the progression of LPS-induced liver injury via the miR-217/TLR4 axis.

## Introduction

Sepsis is a life-threatening condition characterized by a systemic inflammatory response syndrome (SIRS) triggered by a severe infection [1–3]. It accounts for nearly 25%–30% of deaths in intensive care units [4, 5]. The inflammatory cascade in sepsis can cause widespread tissue damage and organ dysfunction, highlighting the critical need for early diagnosis and effective treatment. The liver is particularly vulnerable to sepsis-induced inflammation, with various molecular and cellular processes contributing to liver injury [6, 7]. However, the exact mechanisms behind sepsis-induced liver injury remain poorly understood.

Long non-coding RNAs (lncRNAs), which are over 200 nucleotides in length, have been shown to interact with RNAs, DNAs, and proteins to modulate transcription, chromatin remodeling, and post-transcriptional modification [8]. These lncRNAs play crucial roles in various physiological processes, including cardiovascular diseases, cell cycle regulation, apoptosis, differentiation, and liver disease. Notably, lncRNAs have been implicated in sepsis and lipopolysaccharide (LPS)-induced injury [9, 10]. Aberrant regulation of lncRNAs has been observed in sepsis, with links to organ damage. For instance, lncRNA NEAT1 increases the inflammatory response in sepsis-induced liver injury by regulating the Let-7a/TLR4 (Toll-like receptor 4) signaling pathway [11]. LncRNA MALAT1 modulates sepsis-induced cardiac dysfunction and inflammation through interactions with the p38/MAPK/NF-κB and miR-125b pathways [12]. Additionally, lncRNA H19 acts as a competitive endogenous RNA of aquaporin 1, mediating miR-874 expression in LPS-induced sepsis [13]. LncRNA HOTAIR also elevates tumor necrosis factor-alpha (TNF-α) expression by activating the NF-κB pathway in the cardiomyocytes of an LPS-induced sepsis mouse model [14]. Although lncRNA cancer susceptibility candidate 7 (CASC7) is involved in several disease models, including cancer and spinal cord ischemia-reperfusion injury [15, 16], its role in sepsis remains unknown.

MicroRNAs (miRNAs), which are 20–25 nucleotides in length, play significant roles in regulating gene expression by binding to the 3′-untranslated region (3′-UTR) of mRNAs [17, 18]. These miRNAs are also implicated in the progression of sepsis-induced liver injury. For example, miR-155 exacerbates liver injury by targeting Nrf-2, leading to mitochondrial and endoplasmic reticulum (ER) stress through oxidative stress [19]. Elevated serum miR-122 levels serve as an independent biomarker of liver injury in inflammatory disorders [20], while microRNA-217 (miR-217) is involved in inflammation-related damage [21]. TLR4, a key regulator in various physiological and pathological processes, including liver disease, has been implicated in sepsis-related liver injury [22]. MiR-217 can target TLR4 to modulate podocyte apoptosis [23], but the involvement of the miR-217/TLR4 axis in CASC7-regulated sepsis-induced liver injury is still unclear.

As an endotoxin, LPS interacts with receptors on endothelial cells, leading to acute inflammation [24]. LPS is known to cause sepsis by modulating oxidative stress, inflammatory factors, and endothelial cell growth [25]. In this study, we aimed to investigate and validate the role of CASC7 in LPS-induced liver injury associated with sepsis. We hypothesized that CASC7 might regulate LPS-induced liver injury progression via the miR-217/TLR4 axis. Using a sepsis mouse model created by administering LPS to BALB/c mice, we sought to confirm the functions and mechanisms of CASC7 in LPS-induced liver injury. This study may provide a potential therapeutic target for sepsis-induced liver injury.

## Materials and methods

### Patients and tissue specimens

In this study, 62 sepsis patients with liver injury and 55 healthy controls were enrolled from The First People’s Hospital of Gui Yang between January 2016 and March 2021 (Table 1). Serum samples were collected to detect the expression of CASC7 and miR-217 using q-PCR. The study protocol was approved by the Ethical Committee of The First People’s Hospital of Gui Yang (No. 87645) in accordance with the Helsinki Declaration. Written informed consent was obtained from each patient.

**Table 1:** Clinical characteristics of sepsis patients (*n* ═ 62) and healthy controls (*n* ═ 55)

**Characteristics**	**Healthy controls**	**Patients**	***P* value**
Age (years)	55 ± 12	56 ± 15	0.12
Gender (male, %)	36	43	0.32
BMI (kg/m^2^)	26.33 ± 3.22	26.72 ± 3.13	0.72
WBC count (10^9^/L)	12.45 ± 6.23	17.22 ± 7.23	0.032
CRP (mg/L)	45.26 ± 18.34	68.56 ± 20.56	0.015
Procalcitonin (ng/mL)	7.82 ± 4.11	15.36 ± 6.58	0.008
BUN (mmol/L)	9.32 ± 3.23	19.33 ± 5.32	0.004
Serum creatinine (µM)	78.56 ± 32.54	286.22 ± 89.23	<0.001
Ccr (mL/min 1.73 m^2^)	71.23 ± 13.35	38.22 ± 11.56	<0.001

BMI: Body mass index; WBC: White blood cell; CRP: C-reactive protein; BUN: Blood urea nitrogen; Ccr: Creatinine clearance rate.

### Sepsis mouse model

BALB/c mice were used to establish a sepsis mouse model through LPS treatment. LPS from *Escherichia coli* was obtained from Sigma-Aldrich Co., LLC. (St. Louis, MO, USA). Briefly, four-week-old male BALB/c mice (*n* ═ 25) were housed in a humidity- and temperature-controlled environment with a 12 h/12 h dark/light cycle and free access to water and food. Mice were intraperitoneally injected with LPS (20 mg/kg) or an equal volume of saline. In vitro, LO2 liver cells were treated with 1 µg/mL LPS for varying durations (6, 12, 24, and 48 h), with 12 h chosen for further functional assays. Lentivirus carrying CASC7 shRNA (GenePharma, China) or a corresponding control was injected into the mice via the tail vein. Hematoxylin and Eosin (HE) staining was performed to evaluate liver injury, and ELISA assays were used to measure TNF-α and interleukin 1 beta (IL-1β) expression levels. All procedures and animal care were approved by the Animal Ethics Committee of The First People’s Hospital of Guiyang (No. 67663).

### Cell culture and transfection

LO2 liver cells (human) were purchased from the American Type Tissue Culture Collection. Cells were cultured in DMEM (Gibco, USA) containing 100 units/mL penicillin (Solarbio, China), 0.1 mg/mL streptomycin (Solarbio, China), and 10% fetal bovine serum (Gibco, USA) at 37 ^∘^C with 5% CO_2_. LO2 cells were infected with lentivirus (shCASC7 and shNC, MOI ═ 25) to stably knockdown CASC7. The lentivirus and infection reagent HitransG A were obtained from Genechem (Shanghai, China). After 48 h of incubation with 2 mg/mL puromycin, shCASC7 and shNC LO2 cells were harvested by trypsin digestion and centrifugation. To overexpress CASC7, LO2 cells were infected with a lentivirus system (oe-CASC7 and oe-NC, MOI ═ 50), where oe-NC served as a control. MiR-217 mimic and miRNA NC were provided by Invitrogen (USA). Cell transfection was performed using Lipofectamine 2000 (Invitrogen, USA) according to the manufacturer’s instructions.

### Terminal deoxynucleotidyl transferase dUTP nick end labeling (TUNEL)

A TUNEL detection kit (Roche, Germany) was used to assess cell apoptosis following the manufacturer’s instructions. After TUNEL staining, liver samples were counterstained with DAPI (Sigma, USA) to visualize nuclei. Fluorescence was observed using a confocal microscope (Olympus Fluoview1000, Tokyo, Japan) [28].

### Histology and ELISA analyses

Livers were fixed in 4% paraformaldehyde overnight at room temperature, followed by transfer to 70% ethanol prior to paraffin embedding. Organs were then embedded and frozen using liquid nitrogen-cooled isopentane. Paraffin-embedded samples were sectioned at 4-µm thickness and stained with HE (Nanjing Jiancheng Bioengineering Institute, China) for pathological analysis. Sections were observed under an optical microscope (CKX31SF; Olympus Corporation, Tokyo, Japan). Liver samples were mechanically homogenized in protease-inhibitor (Sigma-Aldrich, USA) containing phosphate-buffered saline, and the homogenates were centrifuged at 11,330 *g* at 4 ^∘^C for 30 min. Protein concentrations of the supernatant fraction were measured using a bicinchoninic acid (BCA) protein measurement kit (R&D Systems, Minneapolis, MN, USA). TNF-α and IL-1β ELISA kits were purchased from R&D Systems. Sheep anti-mouse polyclonal antibody (1:200, MTA00B) was used to detect TNF-α, and mouse monoclonal antibody (1:1000, MLB00C) was used to detect IL-1β [29].

### Cell counting kit-8 (CCK-8) assay

Cell viability was assessed using the CCK-8 assay. Cells (5 × 103) were seeded into 96-well plates and cultured for 12 h. Subsequently, CCK-8 solution (KeyGEN Biotech, China) was added at 0, 24, 48, 72, and 96 h, and the cells were incubated at 37 ^∘^C for an additional 2 h. Absorbance was measured at 450 nm using an ELISA reader (Bio-Tek EL 800, USA) [30].

### Analysis of cell apoptosis

Cell apoptosis was detected by seeding cells into 6-well plates at 2 × 10^5^ cells/well. An Annexin V-FITC Apoptosis Detection Kit (CST, USA) was used to assess apoptosis following the manufacturer’s instructions. Cells were collected, washed with binding buffer (BD Biosciences), stained at 25 ^∘^C, and analyzed by flow cytometry [31].

### Quantitative reverse transcription-PCR (qRT-PCR)

Total RNAs were extracted from mouse liver tissues and cultured cells using TRIZOL (Invitrogen, USA). The first-strand cDNA was synthesized using the cDNA Synthesis Kit (Thermo, USA) according to the manufacturer’s instructions. qRT-PCR was performed using the SYBR Real-time PCR I kit (Takara, Japan), with GAPDH and U6 as internal controls for mRNA and lncRNA, respectively. The primer sequences used are as follows:

CASC7 forward: 5′-ATCAACGTCAAGCTGGGAGG-3′;

CASC7 reverse: 5′-CTTGTCCCCCGCTCGTTC-3′;

TLR4 forward: 5′-TGGATACGTTTCCTTATAAG-3′;

TLR4 reverse: 5′-GAAATGGAGGCACCCCTTC-3′;

MiR-217 forward: 5′-CATGCTCGAGCTTATCAAGGATAAAATACCATG-3′;

MiR-217 reverse: 5′-GTTACGGCCGCTTGAGATCTACTCTAATTTCTTTTTTAAC-3′;

GAPDH forward: 5′-AAGAAGGTGGTGAAGCAGGC-3′;

GAPDH reverse: 5′-TCCACCACCCAGTTGCTGTA-3′;

U6 forward: 5′-GCTTCGGCAGCACATATACTAA-3′;

U6 reverse: 5′-AACGCTTCACGAATTTGCGT-3′.

### Western blot analysis

Total proteins were extracted from liver tissues or cultured cells using RIPA buffer (CST, USA). Protein concentrations were determined using a BCA Protein Quantification Kit (Abbkine, USA). Protein samples were separated using SDS-PAGE (12% polyacrylamide gels) and transferred to PVDF membranes (Millipore, USA). Membranes were blocked with 5% milk and incubated with primary antibodies overnight at 4 ^∘^C. The following primary antibodies were used: PARP TLR4 (1:1000) (Abcam, USA), cleaved PARP (1:1000) (Abcam, USA), caspase-3 (1:1000) (Abcam, USA), cleaved caspase-3 (1:1000) (Abcam, USA), and GAPDH (1:1000) (Abcam, USA) as a loading control. After washing, membranes were incubated with corresponding secondary antibodies (1:1000) (Abcam, USA) at room temperature for 1 h. Images were visualized using an Odyssey CLx Infrared Imaging System, and quantification was performed using ImageJ software [33].

### Luciferase reporter gene assay

The wild-type (WT) and mutant-type (MUT) sequences of CASC7 and TLR4 (with a mutated miR-21 binding site) were amplified and cloned into the PGL4 reporter plasmid. LO2 cells were cotransfected with the fusion plasmid and miR-21 or miR-NC. At 48 h post-transfection, luciferase activity was measured using the Dual Luciferase Assay System (Promega, Madison, WI, USA) following the manufacturer’s instructions [34].

### RNA pull-down assay

Biotin-labeled RNAs were transcribed in vitro using the MEGAscript T7 Kit (Thermo, USA) and purified using the MEGAclear Kit (Thermo, USA) following the manufacturer’s instructions. The biotin-labeled transcripts were then incubated with cell lysates, and streptavidin beads were used to isolate the biotin-labeled RNAs along with any interacting RNAs. The isolated RNAs were then subjected to qPCR analysis [35].

### Ethical statement

This study was conducted in accordance with the ethical guidelines of the Declaration of Helsinki and was approved by the Ethics and Research Committees of The First People’s Hospital of Gui Yang (No. 67663). Consent to participate and consent for publication were not applicable.

### Statistical analysis

All experiments were independently repeated at least three times. Data are presented as mean ± standard deviation (SD). Statistical analysis was performed using GraphPad Prism 7 software. Unpaired Student’s *t*-test was used to compare two groups, while one-way ANOVA was used to compare multiple groups. A *P* value < 0.05 was considered statistically significant.

## Results

### Expression of lncRNA CASC7 and miR-217 in patients with sepsis-induced liver injury

**Figure 1.**
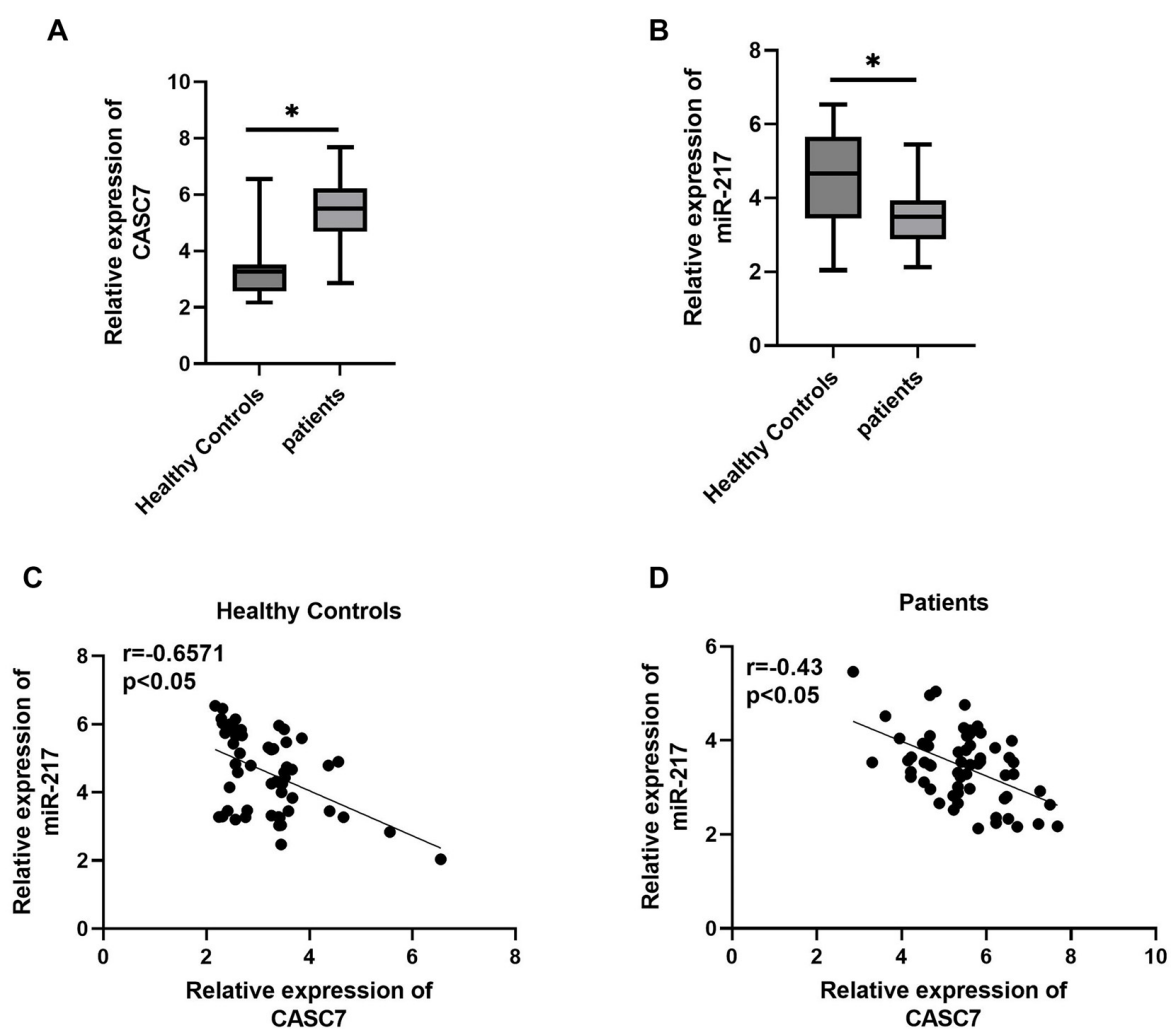
**CASC7 expression was negatively associated with miR-217 in clinical.** The expression of CASC7 (A) and miR-217 (B) was detected in patients with sepsis with liver injury. Then analysis of these CASC7 expression and miR-217 expression in healthy controls (C) and patients with liver injury (D) was identified. *P < 0.05. All experiments were performed in triplicate. CASC7: Cancer susceptibility candidate 7; miR-217: MicroRNA-217.

Compared to healthy controls, the expression of lncRNA CASC7 was significantly increased in sepsis patients with liver injury (Figure 1A), while miR-217 expression was significantly decreased (Figure 1B). Correlation analysis revealed a negative association between CASC7 and miR-217 expression in both healthy controls (Figure 1C) and sepsis patients with liver injury (Figure 1D).

### The expression of CASC7 is positively correlated with LPS-induced liver injury

**Figure 2.**
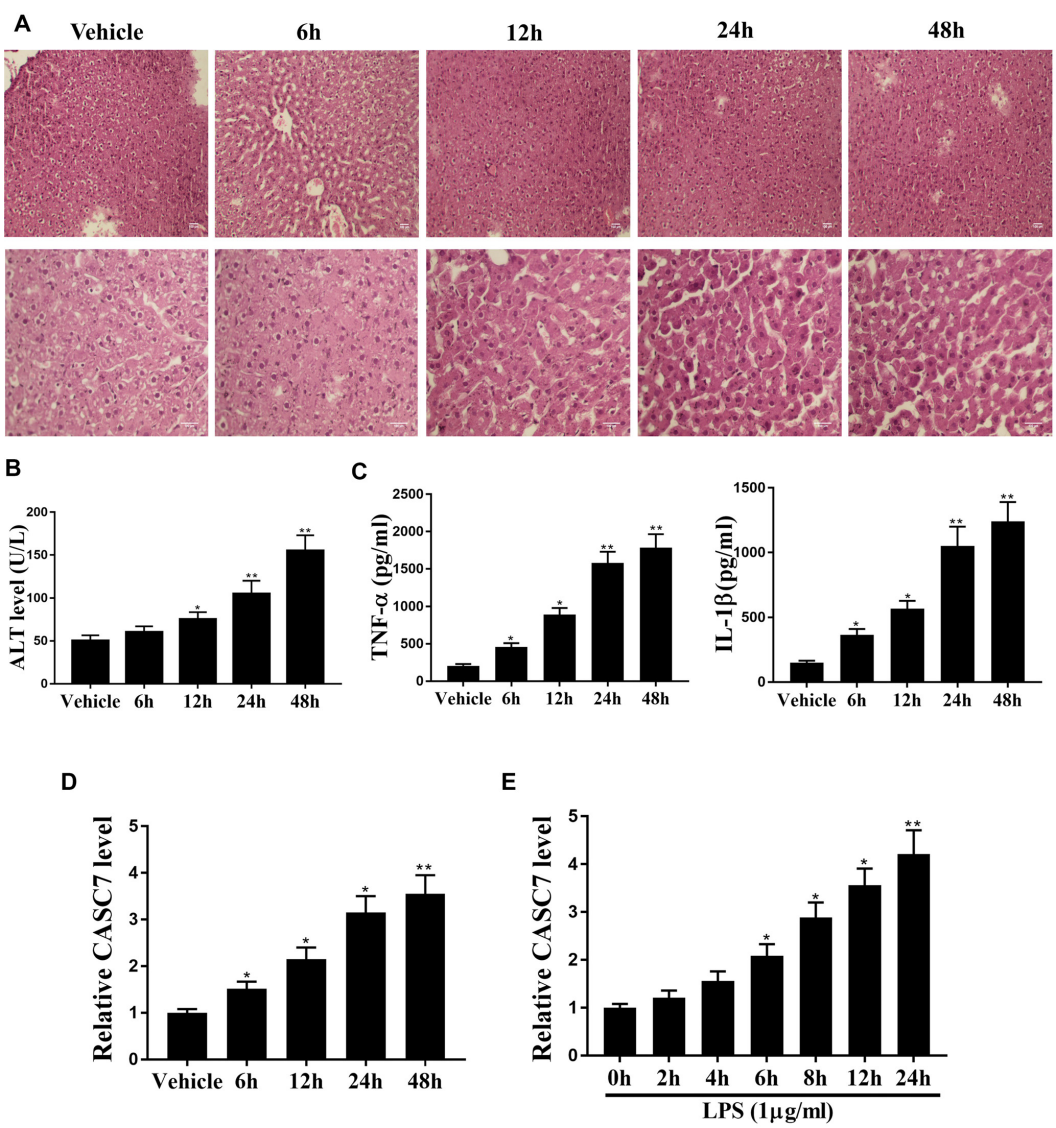
**CASC7 expression is positively correlated with the liver injury induced by sepsis.** (A–C) BALB/c mice were used to establish the sepsis mouse model by the treatment of LPS (LPS concentration ═ 1 µg/mL) (*n* ═ 5). (A) HE staining was used to analyze the liver injury at the indicated time in the mice; (B and C) ALT, TNF-α, and IL-1β levels were measured by ELISA assays at the indicated time in mice; (D) CASC7 expression was tested by qPCR assays at the indicated time in the liver tissues of the mice; (E) The LO2 cells were treated with LPS (1 µg/mL). qPCR assays were used to determine the expression of CASC7 at the indicated time in the cells. Statistic significant differences were indicated: *P < 0.05, **P < 0.01. Data are presented as mean ± SD. All experiments were performed in triplicate. LPS: Lipopolysaccharide; HE: Hematoxylin and Eosin; CASC7: Cancer susceptibility candidate 7; TNF-α: Tumor necrosis factor alpha; IL-1β: Interleukin 1 beta; SD: Standard deviation.

To investigate the role of CASC7 in sepsis-related liver injury, we established a sepsis mouse model via LPS treatment. LPS induced liver injury in a time-dependent manner, as evidenced by liver structural disorder, neutrophil infiltration, cytoplasmic rarefaction, nodular necrosis, and karyopyknosis (Figure 2A). The expression levels of ALT, TNF-α, and IL-1β were also increased in a time-dependent manner (Figure 2B and 2C; *P* < 0.01). Additionally, CASC7 expression was elevated in LPS-treated mouse liver tissues (Figure 2D; *P* < 0.01) and LO2 cells (Figure 2E; *P* < 0.01). These findings suggest a positive correlation between CASC7 expression and LPS-induced liver injury.

### Knockdown of CASC7 relieves LPS-induced liver injury in vivo

**Figure 3.**
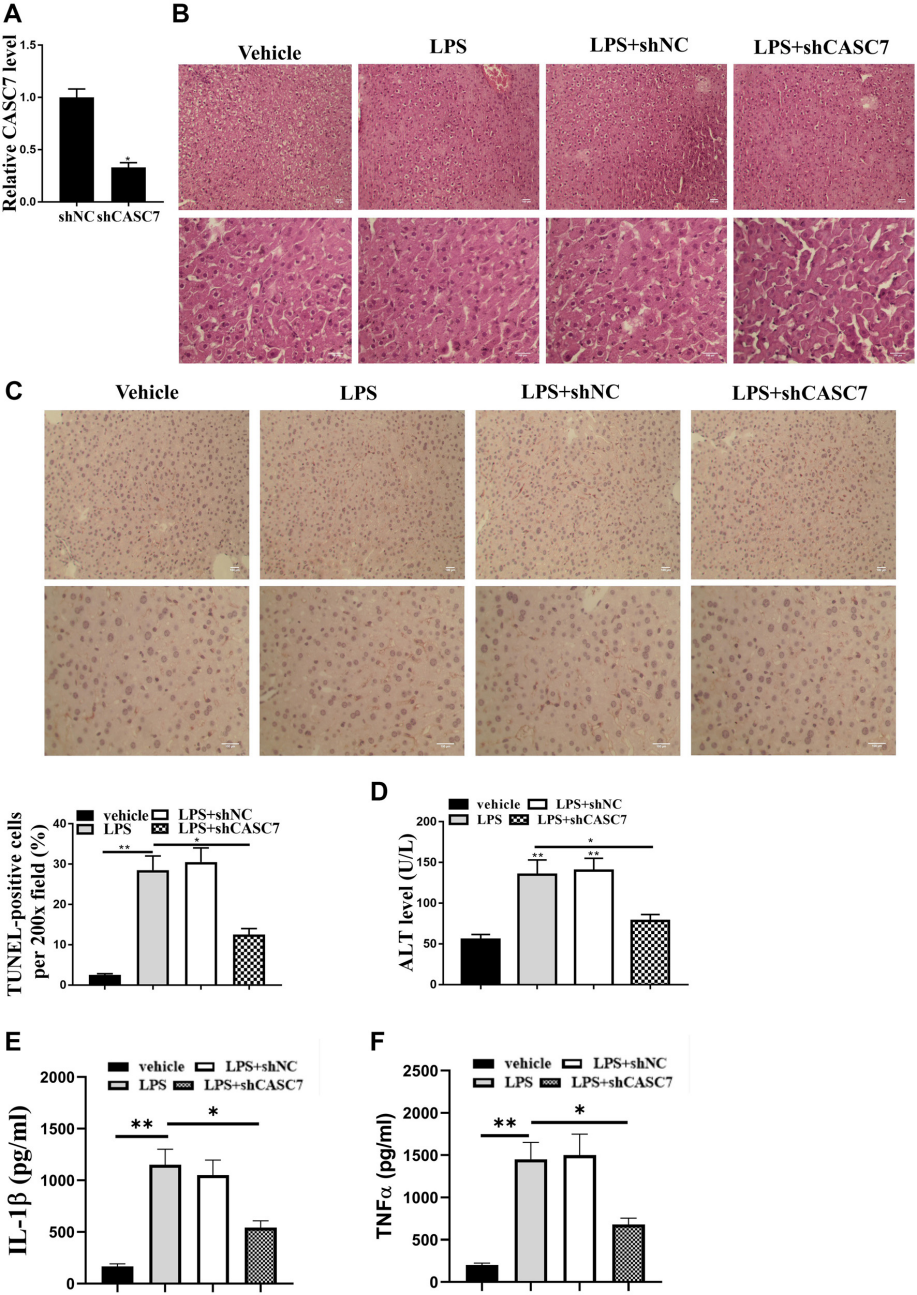
**CASC7 depletion relieves sepsis-induced liver injury in vivo.** (A–D) LPS or co-treated with LPS and control shRNA or CASC7 shRNA were used to treat BALB/c mice. (A) CASC7 expression was tested by qPCR assays at the indicated time in the liver tissues of the mice; (B) HE staining was used to analyze the liver injury in mice; (C) TUNEL analysis was used to measure the apoptosis in liver tissues of mice; (D and E) ALT, (E) TNF-α, and (F) IL-1β levels were measured by the ELISA assays in the mice. Statistic significant differences were indicated: *P < 0.05, **P < 0.01. Data are presented as mean ± SD. All experiments were performed in triplicate. CASC7: Cancer susceptibility candidate 7; LPS: Lipopolysaccharide; HE: Hematoxylin and eosin; TNF-α: Tumor necrosis factor alpha; IL-1β: Interleukin 1 beta; TUNEL: Transferase dUTP nick end labeling; SD: Standard deviation; shRNA: Short hairpin RNA.

Next, we examined the effect of CASC7 on LPS-induced liver injury progression in vivo. Specific short hairpin RNAs (shRNAs) against CASC7 were used to knock down its expression. Among three CASC7 shRNAs tested, sh-CASC7#1 significantly downregulated CASC7 expression (Figure 3A; *P* < 0.05). Knockdown of CASC7 markedly decreased LPS-induced liver injury in mice (Figure 3B). TUNEL assay showed that LPS treatment increased TUNEL-positive cell numbers, but CASC7 knockdown blocked this increase, indicating reduced liver injury (Figure 3C; *P* < 0.05). Furthermore, CASC7 knockdown inhibited LPS-induced elevations in ALT (Figure 3D), TNF-α (Figure 3E), and IL-1β (Figure 3F) levels in mice (*P* < 0.05). These results suggest that CASC7 knockdown alleviates LPS-induced liver injury in vivo.

**Figure 4.**
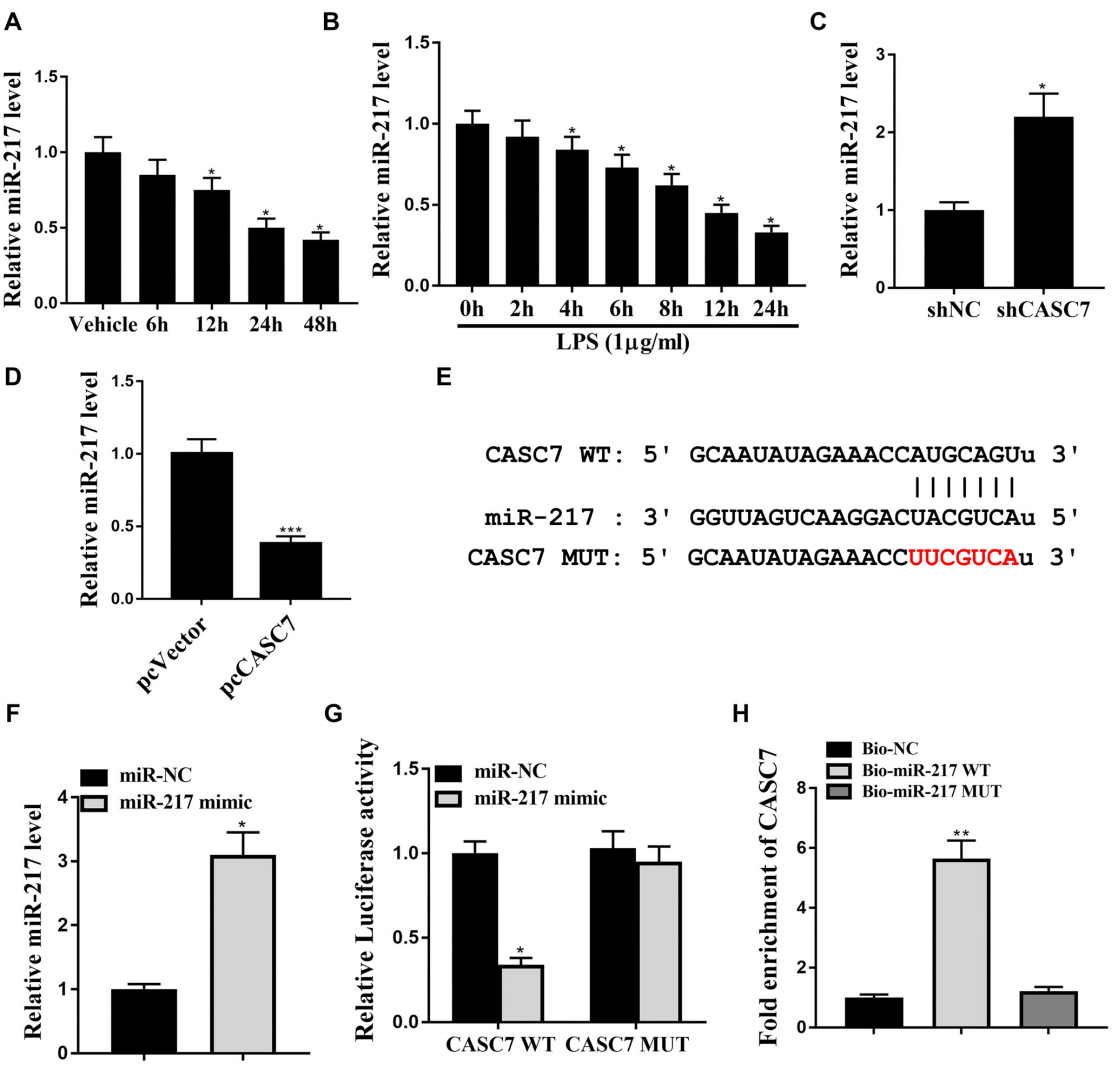
**CASC7 targets miR-217 in liver cells.** (A) BALB/c mice were used to establish the sepsis mouse model by the treatment of LPS (20 mg/kg) (*n* ═ 5). MiR-217 expression was tested by qPCR assays at indicated time in liver tissues of mice. (B) LPS (1 µg/mL) was used to treat the LO2 cells. MiR-217 expression was determined by qPCR assays at the indicated time in the cells. (C) Control shRNA or CASC7 shRNA were transfected into LO2 cells. MiR-217 expression was assessed by qPCR assays. (D) MiR-217 expression was detected in LO2 cells transfected with pcCASC7. (E) Using Starbase 3.0v software, the potential interaction between CASC7 and miR-217 was identified. (F) MiR-217 expression was determined by qPCR assays. (G) The luciferase activities of wild type CASC7 (CASC7 WT), and CASC7 with the miR-217-binding site mutant (CASC7 MUT). (H) RNA pull-down assay was used to analyze the interaction of CASC7 and miR-217. Statistic significant differences were indicated: * *P* < 0.05, **P < 0.01. Data are presented as mean ± SD. All experiments were performed in triplicate. LPS: Lipopolysaccharide; CASC7: Cancer susceptibility candidate 7; miR-217: MicroRNA-217; SD: Standard deviation; shRNA: Short hairpin RNA.

### MiR-217 is a target of CASC7

We further explored the mechanism by which CASC7 mediates liver injury induced by LPS. The expression levels of miR-217 were reduced by LPS treatment in a time-dependent manner in mouse liver tissues (Figure 4A; *P* < 0.05) and LO2 cells (Figure 4B; *P* < 0.05). Meanwhile, the knockdown of CASC7 enhanced (*P* < 0.05), and overexpression of CASC7 inhibited (*P* < 0.001) the expression of miR-217 in LO2 cells (Figure 4C and 4D). The potential interaction between CASC7 and miR-217 was predicted using Starbase 3.0v software (Figure 4E). The efficiency of the miR-217 mimic was confirmed in LO2 cells (Figure 4F; *P* < 0.05). The miR-217 mimic attenuated the luciferase activity of CASC7, but not CASC7 with a mutated miR-217 binding site in LO2 cells (Figure 4G; *P* < 0.05). RNA pull-down assays further confirmed that wild-type Bio-miR-217, but not mutant Bio-miR-217, could interact with CASC7 (Figure 4H; *P* < 0.01). These results suggest that miR-217 is a direct target of CASC7.

**Figure 5.**
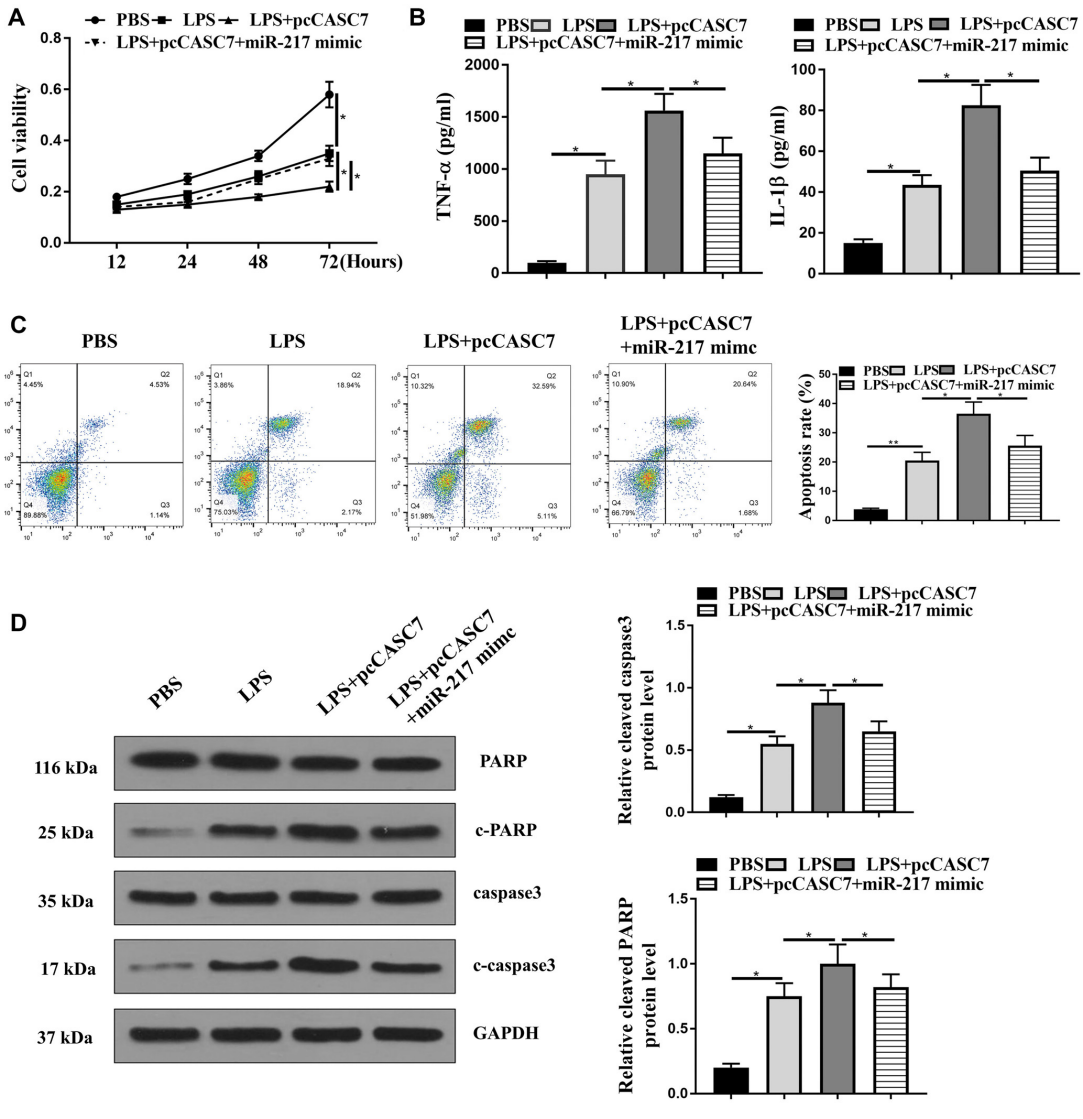
**CASC7 promotes LPS-induced liver injury progression by targeting miR-217.** (A–D) The LO2 cells were co-treated with LPS (1 µg/mL) and CASC7 overexpression vector, or LPS (1 µg/mL), or co-treated with LPS (1 µg/mL), CASC7 overexpression vector, and miR-217 mimic. (A) CCK-8 assay was used to measure the cell viability; (B) TNF-α and IL-1β levels were measured by the ELISA assays in the cells; (C) Flow cytometry was used to measure cell apoptosis; (D) PARP, cleaved PARP (c-PARP), caspase3, cleaved caspase3 (c-caspase3), and GAPDH expression was evaluated by Western blot. Statistic significant differences were indicated: *P < 0.05, **P < 0.01. Data are presented as mean ± SD. All experiments were performed in triplicate. CASC7: Cancer susceptibility candidate 7; miR-217: MicroRNA-217; LPS: Lipopolysaccharide; CCK-8: Cell counting kit-8; TNF-α: Tumor necrosis factor alpha; IL-1β: Interleukin 1 beta; SD: Standard deviation.

**Figure 6.**
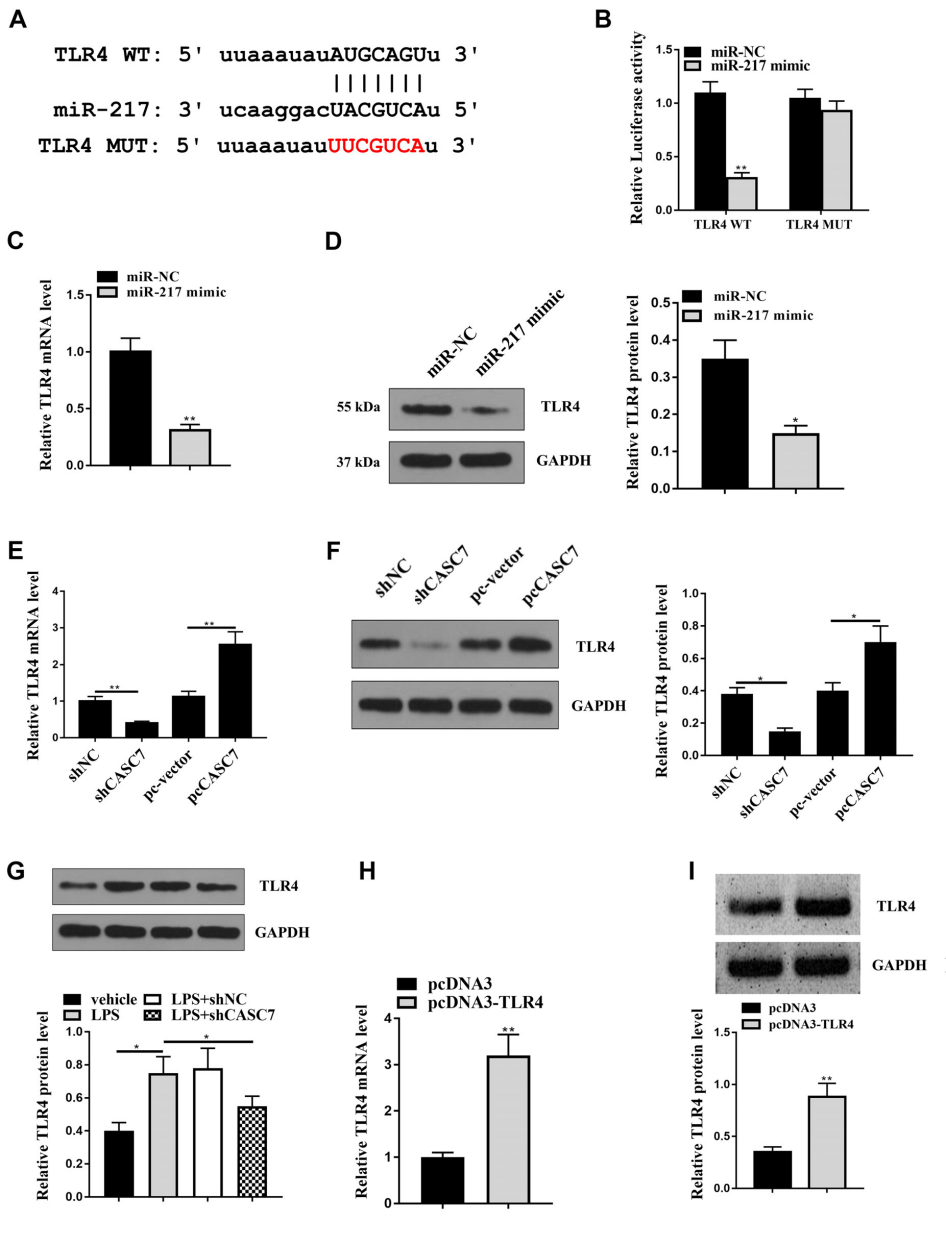
**MiR-217 targets TLR4 in liver cells, and TLR4 expression could be regulated by CASC7 or MiR-217.** (A) Using miRDB and miRmap software, the interaction of miR-217 and TLR4 3’ UTR was identified; (B and C) The LO2 cells were treated with control mimic or miR-217 mimic; (B) The luciferase activities of TLR4 with the miR-217-binding site mutant (TLR4 MUT) and wild type TLR4 (TLR4 WT) were determined in cells; (C and D) TLR4 expression was measured by qRT-PCR and western blot assay in cells; (E and F) LO2 cells were treated CASC7 shRNA, CASC7 overexpression vector, or the corresponding control. TLR4 expression was tested by western blot and qRT-PCR; (G) LPS (1 µg/mL), co-treated with LPS (1 µg/mL) and control shRNA or CASC7 shRNA were transfected into LO2 cells. TLR4 expression was assessed by western blot and qRT-PCR; (H and I) LO2 cells were transfected with TLR4 overexpression vector or control vector. western blot and qRT-PCR assay were used to measure TLR4 expression. Statistic significant differences were indicated: *P < 0.05, **P < 0.01. Data are presented as mean ± SD. All experiments were performed in triplicate. CASC7: Cancer susceptibility candidate 7; miR-217: MicroRNA-217; LPS: Lipopolysaccharide; TLR4: Toll-like receptor 4; 3’-UTR: 3’-untranslated region; SD: Standard deviation; shRNA: Short hairpin RNA.

### CASC7 promotes LPS-induced liver injury progression by targeting miR-217

Next, we assessed whether CASC7 promotes LPS-induced liver injury progression by targeting miR-217 in liver cells. Overexpression of CASC7 inhibited cell viability, while the miR-217 mimic rescued cell viability in LPS-treated LO2 cells (Figure 5A; *P* < 0.05). miR-217 mimic treatment also reversed CASC7 overexpression-enhanced expression levels of IL-1β and TNF-α in LPS-treated LO2 cells (Figure 5B; *P* < 0.05). Additionally, CASC7 overexpression-induced apoptosis was reduced by miR-217 mimic treatment in LPS-treated LO2 cells (*P* < 0.05) (Figure 5C). The elevated expression of cleaved caspase-3 (c-caspase-3) and cleaved PARP (c-PARP) by CASC7 overexpression was similarly reduced by miR-217 mimic treatment in LPS-treated LO2 cells (Figure 5D; *P* < 0.05). These results indicate that CASC7 promotes LPS-induced liver injury progression by targeting miR-217 in vitro.

**Figure 7.**
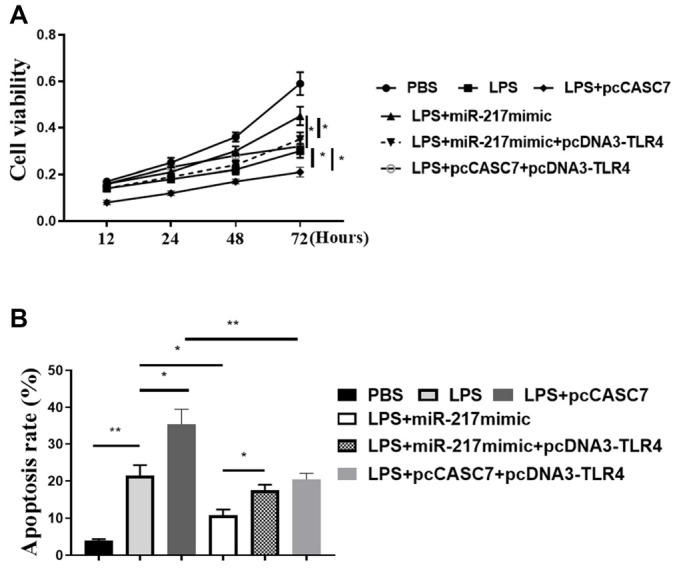
**TLR4 could reversed the miR-217 mimic-increased or CASC7 decreased function in liver cells.** (A) CCK-8 assay was used to measure cell viability; (B) Flow cytometry was used to measure cell apoptosis. Statistic significant differences were indicated: *P < 0.05, **P < 0.01. Data are presented as mean ± SD. All experiments were performed in triplicate. CASC7: Cancer susceptibility candidate 7; miR-217: MicroRNA-217; CCK-8: Cell counting kit-8; TLR4: Toll-like receptor 4; SD: Standard deviation.

### MiR-217 attenuates liver injury induced by CASC7 by targeting TLR4 in vitro

To explore the downstream targets of miR-217, we identified a miR-217 binding site in the 3′-UTR of TLR4 (Figure 6A). The miR-217 mimic attenuated TLR4 luciferase activity but not the luciferase activity of TLR4 with a mutated miR-217 binding site in LO2 cells (Figure 6B; *P* < 0.01). Overexpression of miR-217 significantly reduced TLR4 expression at both mRNA (*P* < 0.01) and protein (*P* < 0.05) levels (Figure 6C and 6D). CASC7 knockdown downregulated, while CASC7 overexpression upregulated TLR4 expression at both mRNA (*P* < 0.01) and protein (*P* < 0.05) levels in vitro (Figure 6E and 6F). CASC7 knockdown also reduced LPS-induced TLR4 expression in vitro (Figure 6G and 6I; *P* < 0.01). The efficiency of TLR4 overexpression was validated in vitro (Figure 6H; *P* < 0.01). TLR4 overexpression blocked miR-217 mimic-increased or CASC7-decreased LO2 cell viability (Figure 7A; *P* < 0.05) and enhanced cell apoptosis (Figure 7B; *P* < 0.05). These results suggest that miR-217 attenuates CASC7-induced liver injury by targeting TLR4 in vitro.

## Discussion

Sepsis is a systemic inflammatory disease caused by infections, severe trauma, or burns, leading to multiple organ failure, including liver injury [36]. The incidence and mortality of sepsis-induced liver injury are high [6]. Despite extensive research, the underlying mechanisms of sepsis-induced liver injury remain elusive. In this study, we found that CASC7 contributes to LPS-induced liver injury progression by modulating the miR-217/TLR4 axis.

Multiple lncRNAs have been implicated in the development of sepsis-induced liver injury. For example, lncRNA colorectal neoplasia differentially expressed (CRNDE) alleviates sepsis-caused liver damage by targeting miR-126-5p [37]. Circulating lncRNA NEAT1 is associated with an unfavorable prognosis and increased risk in sepsis patients [38]. lncRNA SNHG16 mediates the effects of miR-15a/16 on the LPS-induced inflammatory pathway [39]. LPS promotes sepsis progression by activating the HULC/miR-204-5p/TRPM7 axis in HUVECs [40]. lncRNA TapSAKI exacerbates inflammatory injury and sepsis-induced kidney injury [41]. lncRNA HOTAIR has been reported to mediate severe organ injury in a sepsis rat model through the miR-34a/Bcl-2 axis [42]. lncRNA NEAT1 plays a crucial role in sepsis-induced severe injury by modulating miR-204 and the NF-κB signaling pathway [43]. Enhanced expression of lncRNAs HULC and UCA1 is required for the proinflammatory response in LPS-induced endothelial cell sepsis [44]. Previous studies have shown that hydrogen sulfide upregulates lncRNA CASC7 to reduce neuronal cell apoptosis in a spinal cord ischemia-reperfusion injury rat model [16]. In the present study, we identified that CASC7 expression was elevated in LPS-treated mice, suggesting that CASC7 is involved in LPS-induced liver injury.

As key regulators of pathological and physiological processes, miRNAs also play a significant role in modulating sepsis-induced liver injury. MiR-155, for instance, regulates the JAK/STAT signaling pathway and mediates sepsis-induced liver damage [45]. Paclitaxel alleviates septic liver damage by modulating the miR-27a/TAB3/NF-κB axis [46]. MCPIP1 reduces LPS-induced liver injury by regulating SIRT1 expression via miR-9 [47]. MiR-103a-3p inhibits sepsis-induced liver injury by targeting high-mobility group box protein B1 (HMGB1) [48]. MiR-21 is also involved in sepsis [49], while miR-195 inhibits multiple organ injury and apoptosis in sepsis mouse models [50]. MiR-30e suppresses apoptosis and promotes hepatocyte proliferation in cecal ligation and puncture-induced sepsis by regulating the JAK/STAT signaling mediated by FOSL2 [51]. Puncture and cecal ligation-induced sepsis is correlated with the inhibited expression of adenylyl cyclase nine and enhanced expression of miR-142-3p [52]. Our data demonstrate that CASC7 targets miR-217 in liver cells and promotes LPS-induced liver injury progression by sponging miR-217. These findings provide valuable insights into the role of miR-217 in CASC7-mediated liver injury progression induced by LPS.

TLR4 is another critical player in sepsis-induced liver injury. TLR4 antagonists, such as eritoran tetrasodium, inhibit hepatic ischemia-reperfusion injury by blocking the HMGB1 signaling pathway [53]. Dexmedetomidine alleviates septic liver damage by downregulating TLR4 and the MyD88/NF-κB signaling pathway [54]. Trichostatin A protects the liver from septic injury by inhibiting TLR4 signaling [55]. Green tea extract restores liver metabolism, likely through its anti-inflammatory effects mediated by the endotoxemia/TLR4/NF-κB pathway [56]. Leukoadhesin-1 protects against endotoxic shock in mice by inhibiting LPS-TLR4 interactions [57]. Our study reveals that miR-217 attenuates sepsis-induced liver injury by targeting TLR4, suggesting that TLR4 is involved in the modulation of LPS-induced liver injury. Furthermore, both CASC7 and miR-217 were found to regulate TLR4 expression in vitro, and TLR4 overexpression reversed the effects of CASC7/miR-217 in vitro.

## Conclusion

We found that CASC7 contributes to sepsis-induced liver injury progression by targeting the miR-217/TLR4 axis. CASC7, miR-217, and TLR4 may serve as potential therapeutic targets for LPS-induced liver injury. Our results help expand the understanding of the molecular mechanisms involved in sepsis.

## Data Availability

The datasets used and analyzed during the current study are available from the corresponding author on reasonable request.
